# Phenotyping viral infection in sweetpotato using a high-throughput chlorophyll fluorescence and thermal imaging platform

**DOI:** 10.1186/s13007-019-0501-1

**Published:** 2019-10-22

**Authors:** Linping Wang, Sylvain Poque, Jari P. T. Valkonen

**Affiliations:** Department of Agricultural Sciences, University of Helsinki, P.O. Box 27, 00014 Helsinki, Finland

**Keywords:** High-throughput phenotyping, Chlorophyll fluorescence imaging, Thermal infrared imaging, Sweetpotato, Virus co-infection, SPCSV, SPFMV, Gene expression

## Abstract

**Background:**

Virus diseases caused by co-infection with *Sweet potato feathery mottle virus* (SPFMV) and *Sweetpotato chlorotic stunt virus* (SPCSV) are a severe problem in the production of sweetpotato (*Ipomoea batatas* L.). Traditional molecular virus detection methods include nucleic acid-based and serological tests. In this study, we aimed to validate the use of a non-destructive imaging-based plant phenotype platform to study plant-virus synergism in sweetpotato by comparing four virus treatments with two healthy controls.

**Results:**

By monitoring physiological and morphological effects of viral infection in sweetpotato over 29 days, we quantified photosynthetic performance from chlorophyll fluorescence (ChlF) imaging and leaf thermography from thermal infrared (TIR) imaging among sweetpotatoes. Moreover, the differences among different treatments observed from ChlF and TIR imaging were related to virus accumulation and distribution in sweetpotato. These findings were further validated at the molecular level by related gene expression in both photosynthesis and carbon fixation pathways.

**Conclusion:**

Our study validated for the first time the use of ChlF- and TIR-based imaging systems to distinguish the severity of virus diseases related to SPFMV and SPCSV in sweetpotato. In addition, we demonstrated that the operating efficiency of PSII and photochemical quenching were the most sensitive parameters for the quantification of virus effects compared with maximum quantum efficiency, non-photochemical quenching, and leaf temperature.

## Background

Sweetpotato (*Ipomoea batatas* L.) is one of the most important staple food crops in the world [1]. Plant viruses are the most harmful pathogen of this crop, of which aphid-transmitted *Sweet potato feathery mottle virus* (SPFMV, genus *Potyvirus*) is the most widespread virus that infects sweetpotatoes. In addition, the whitefly-transmitted, phloem-limited *Sweet potato chlorotic stunt virus* (SPCSV, genus *Crinivirus*) is problematic because of its synergistic interaction with many other viruses [2–5]. Among these synergisms, sweetpotato virus disease caused by the co-infection of SPCSV and SPFMV is the most devastating in sweetpotato [6, 7]. Previous studies on this synergism demonstrate that the protein RNase III encoded by SPCSV is able to break down the plant’s antiviral resistance, which is based on RNA silencing [8]. Sweetpotato plants co-infected with SPCSV and SPFMV commonly display leaf deformation, mosaic symptoms, yellowing, vein clearing, dwarfing, and stunting [9]. Along with these severe symptoms, co-infection with SPCSV and SPFMV causes a reduction in chlorophyll content and thus in the photosynthetically active radiation [10].

Traditional molecular methods, including serological and nucleic acid-based approaches, have been used to establish virus diagnosis. Imaging-based plant disease detection methods have become low cost, rely on equipments, and less labour intensive and time consuming as compared with traditional methods [11, 12]. Thus, it is likely that they will play an important role in future precision farming and pest management. Moreover, the development of image capture techniques and the availability of more open-source software allow the generation of time-series profiles in plants with respect to their photosynthesis performance, stress situation, and plant–pathogen interactions in a robust and non-destructive way [13–16]. With imaging-based methods, the impact of viruses on plant photosynthesis and transpiration can be monitored by chlorophyll fluorescence (ChlF) of PSII and thermal infrared (TIR) imaging [17]. For example, *Soybean mosaic virus* (SMV) in soybean causes depression of the effective quantum yield of PSII (ФPSII) [18]. A temperature increase caused by *Tobacco mosaic virus* (TMV) or *Potato virus Y* (PVY) infection can be detected in pre-symptomatic leaves by thermal imaging [19, 20]. A recent study showed that viral infection alter plant transcriptome and noted that the maximum quantum yield (QY_max) of sweetpotato co-infected with SPFMV, *Sweetpotato virus 2* (SPV2), and *Sweetpotato virus G* (SPVG) differed significantly from that of healthy plants [21].

The National Plant Phenotyping Infrastructure (NaPPI) platform at Viikki campus, University of Helsinki, allows us to systemically monitor dynamic interactions between viral treatments and plant development. Specifically, morphological and physiological traits can be monitored by RGB imaging, photosynthesis performance by ChlF imaging, and leaf temperature by TIR imaging [22]. To study plant stress, the most informative parameters from ChlF imaging are minimal ChlF yield (F0), maximum ChlF yield (Fm), variable fluorescence (Fv), QY_max, ФPSII, photochemical quenching (qP), and non-photochemical quenching (NPQ) among other characteristics [11, 23]. At the molecular level, genes that encode proteins of the photosynthesis complex (*PsbA*, *PsbC*, *PsbE*, and *PsaA*) and photosynthesis regulators (such as *PsbN*) are globally downregulated under biotic stress in plants [24]. Moreover, modulation of Rubisco expression under stress conditions correlates with a decrease in PSII efficiency caused by stomatal closure [25–28].

Here we used physiological and morphological parameters, including plant height, biomass, leaf surface area, and allocation of shoot/root ratio to investigate different viral impacts on sweetpotato growth. In addition, the viral effect on both photosynthesis performance and stress indicators was monitored by ChlF and TIR imaging, respectively. To investigate consistency of the viral effect under different conditions, two experiments consisting of six viral treatments were carried out under two growth conditions. Finally, virus accumulation and distribution in plants, and host photosynthesis-related gene expression were quantified by real-time quantitative PCR (RT-qPCR) to understand the interactions between plant’s phenotyping and virus infection. Analysing the correlation between viral treatments and plant growth performance is expected to provide crucial information about imaging-based methods and remote sensing technologies to efficiently detect viral infections for scientific experiments in greenhouses and for precision agriculture in the field.

## Results

### Effect of viral infection on plant development

Viral diseases are typically associated with morphological and physiological changes in plants that affect their biomass and height. To compare the plant growth process among sweetpotato plants from six treatments, we monitored plant height for 29 days and biomass after 31 dpt under two growth conditions.

### Effect on growth

**Fig. 1:**
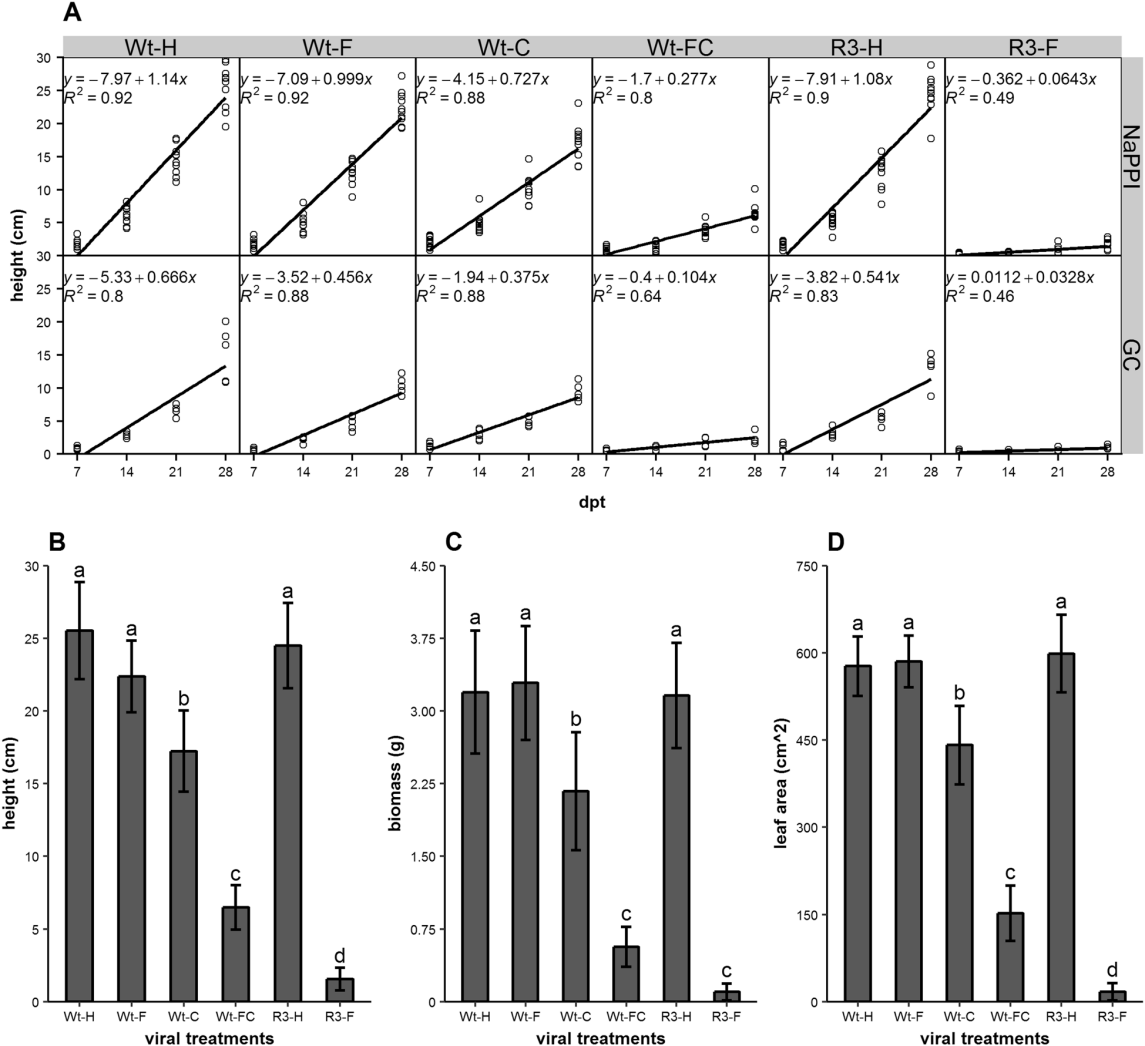
Effects of viral infection and growth conditions on plant growth. **A** Linear regression model of plant height over time. Plant height was measured after 7, 14, 21, and 28 dpt. Wild-type sweetpotato was either healthy (Wt-H) or infected with SPFMV (Wt-F), SPCSV (Wt-C), or both viruses (Wt-FC). Transgenic sweetpotato expressing RNase III from SPCSV was either healthy (R3-H) or infected with SPFMV (R3-F). All six conditions were assessed in plants growing in the growth chamber (GC) and at the NaPPI facility (NaPPI). **B**–**D** Height (**B**), biomass (**C**), and total leaf area (**D**) of plants growing at NaPPI were measured at 31 dpt. Data are shown as the mean ± SE (n = 10). Different letters indicate statistically significant differences (Tukey’s HSD test, P < 0.05)

Viral effects of SPFMV and SPCSV on sweetpotato development were characterized by monitoring plants grown in two separate facilities, the NaPPI and a growth chamber, which differed only in their light intensity (260 and 60–70 µmol m^−2^ s^−1^, respectively). The growth conditions had an effect on plant growth rate represented by plant height (P < 0.001, ANOVA). We observed an average decrease in height of 51% between plants in the NaPPI facility and growth chamber: 42% for wild-type healthy sweetpotato (Wt-H), 54% for sweetpotato infected with SPFMV (Wt-F), 48% for sweetpotato infected with SPCSV (Wt-C), 62% for sweetpotato infected with both SPFMV and SPCSV (Wt-FC), 50% for healthy RNase III transgenic sweetpotato (R3-H); and 49% for RNase III transgenic sweetpotato infected with SPFMV (R3-F) (Fig. 1A).

Thereafter, differences among the six treatments only for plants grown under the same conditions were analysed. For NaPPI-grown plants, plant height did not show significant differences among non-infected plants (Wt-H and R3-H) and plants infected only with SPFMV (Wt-F). Compared with those three treatments (average of plant height, 24.1 cm), a significant reduction was observed in plants infected with SPCSV (Wt-C, 17.2 cm) and co-infected with SPFMV and SPCSV (Wt-FC, 6.5 cm) and in RNase III transgenic plants infected with SPFMV (R3-F, 1.6 cm) (Fig. 1B) (P < 0.001). In addition, total biomass and leaf surface area showed the similar pattern (Fig. 1C, D), but there was no statistical difference between Wt-FC and R3-F in biomass (Fig. 1C). Thus, SPCSV single infection (Wt-C), SPFMV and SPCSV co-infection (Wt-FC), and SPFMV infection of transgenic plants (R3-F) had severe impacts on sweetpotato growth. Full-length images of six virus-infected plants under the two growth conditions are shown in Additional file 1: Fig. S1.

### Effect of biomass allocation

**Fig. 2:**
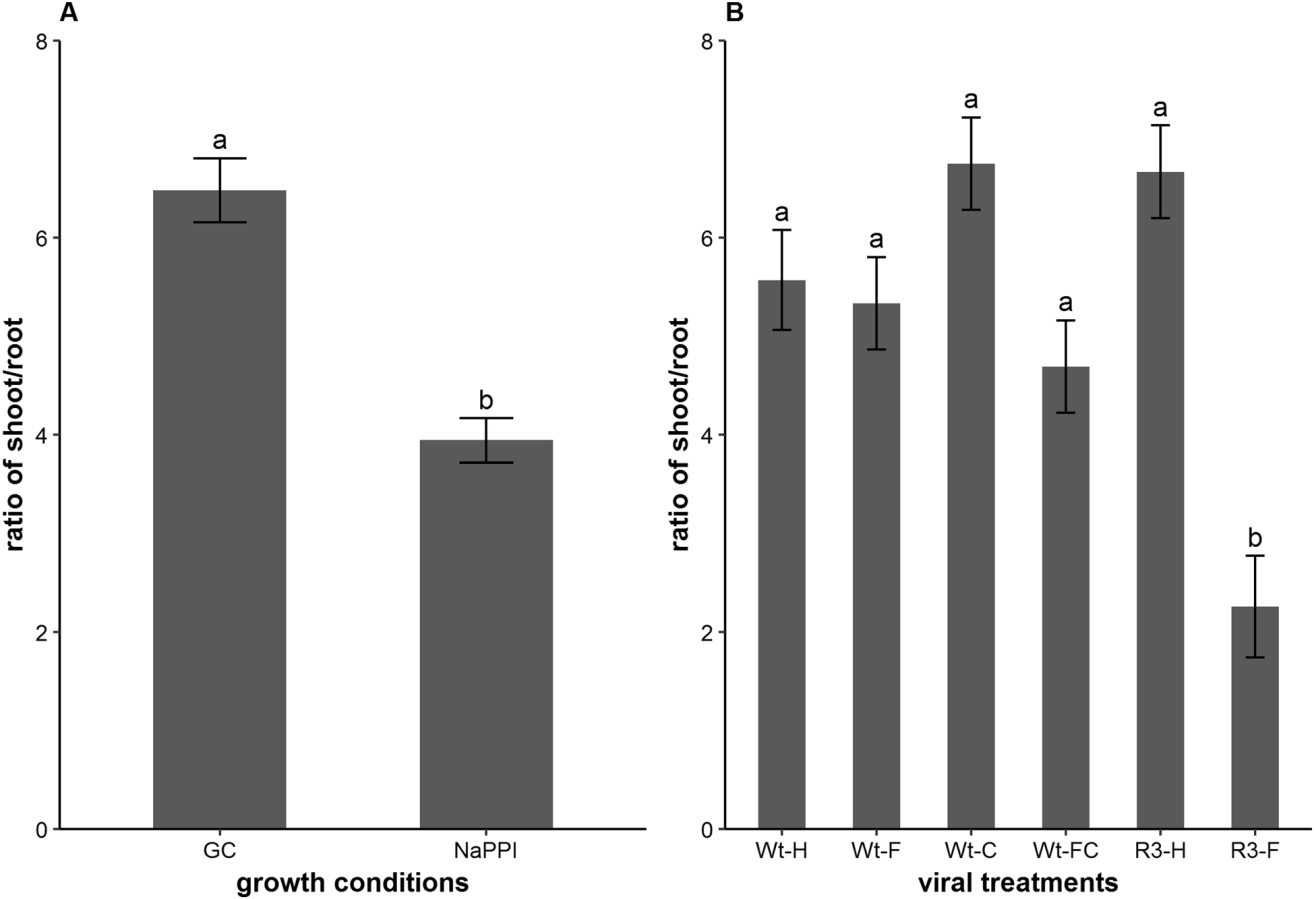
Biomass of shoot/root ratio of the six viral treatments under two growth conditions. **A** Global shoot/root ratio of wild-type and transgenic sweetpotato grown in the growth chamber (GC) or NaPPI facility (n = 30 and 60, respectively). **B** Ratio of shoot/root for wild-type and transgenic sweetpotato plants (n = 13–15) grown at the NaPPI facility and differentially treated. Measurements were made at 31 dpt and expressed as the mean ± SE. Different letters indicate statistically significant differences (Tukey’s HSD test, P < 0.05)

Considering the different functionality of leaves (carbon fixation, i.e., photosynthesis), stems (structural support and transport), and roots (nutrient and water absorption) in plants [29, 30], we decided to complement growth and morphological analysis with the determination of biomass allocation. Two factors, viral treatment and growth condition, and the interaction between viral treatments and growth condition had significant contributions to the differential shoot/root biomass ratio (P < 0.001, Levene’s Test). The results showed that plants under growth chamber conditions had a significantly higher shoot/root ratio (6.48) than did plants from NaPPI (3.94) (P < 0.001) (Fig. 2A), which was most likely due to the weaker light intensity in the growth chamber. However, the mean ratios from six viral treatments indicated that there were no significant differences among them (average 5.19), except for RNase III transgenic plants infected with SPFMV (R3-F, 2.56); these plants had the most severe disease symptoms according to the scoring index of Mwanga et al. [31], and developed a relatively large roots as compared with the other five treatments (Fig. 2B).

### Virus distribution and titres

**Fig. 3:**
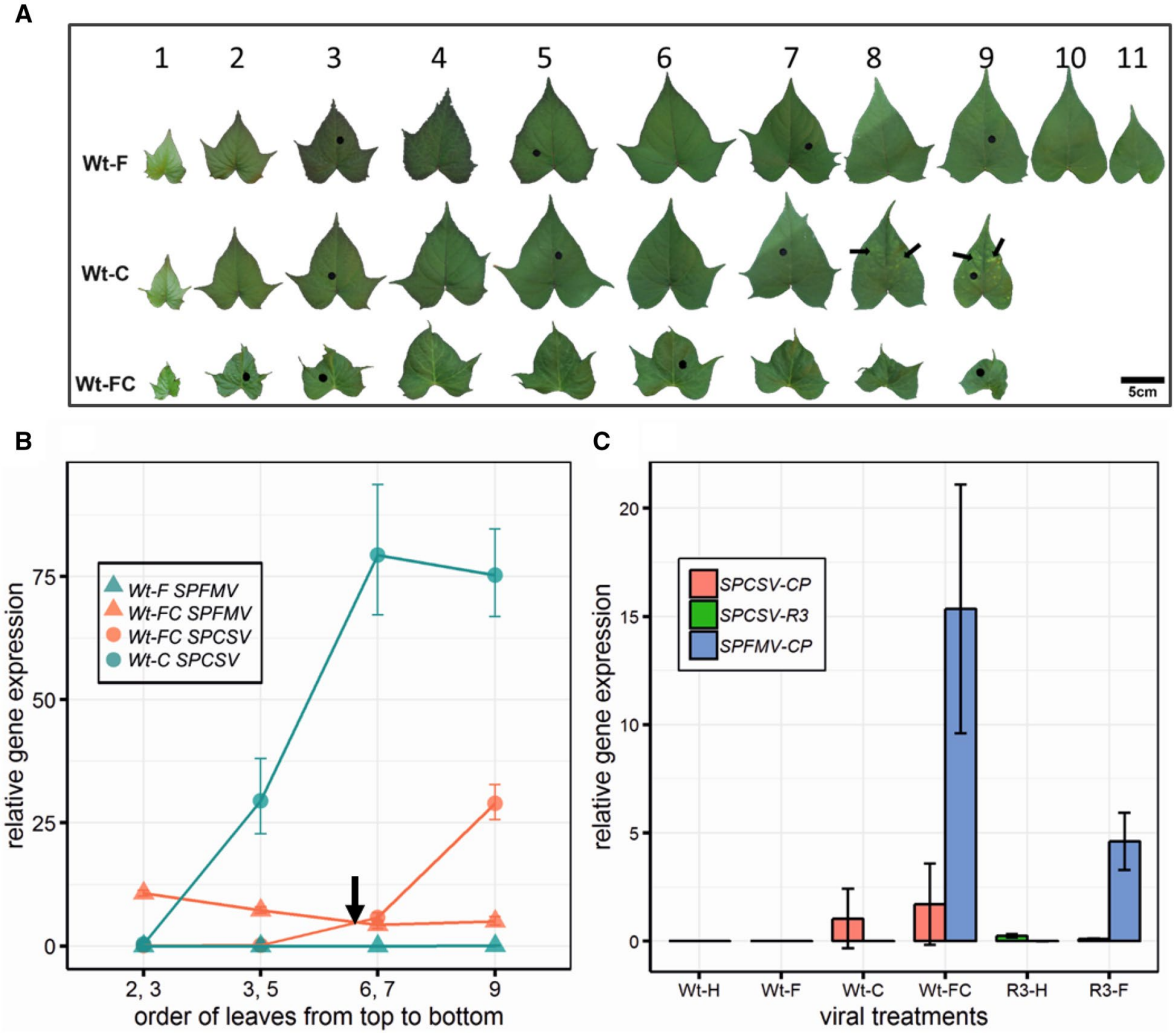
Characterization of viral infection, distribution, and accumulation in plants grown at NaPPI. **a** Detached leaves from wild-type sweetpotato plants infected with only SPFMV (Wt-F) or SPCSV (Wt-C) or co-infected with SPFMV and SPCSV (Wt-FC) at 31 dpt. Numbers above the leaves indicate leaf order on the plant from top to bottom. Black arrows indicate typical mosaic symptoms on older leaves of Wt-C plants, whereas leaf deformation and vein clearing can be observed on almost all leaves of Wt-FC plants. Holes (black circles) on leaves correspond to the sampling regions for the viral accumulation assay. **B** SPCSV and SPFMV distribution from top to bottom leaves in Wt-F, Wt-C, and Wt-FC sweetpotato plants. Leaf numbering along the *x* axis corresponds to the sampling leaf order in **A**. The black arrow indicates the cross-over point around leaf five between SPCSV and SPFMV localization in Wt-FC plants. Data are expressed as the mean ± SD from two sample pools representing four plants in total. **C** Relative quantification of viral accumulation among all six sweetpotato treatment groups. Viral accumulation was estimated at 31 dpt in plants grown at NaPPI by measuring the relative gene expression of viral coat protein (SPFMV-CP and SPCSV-CP) and RNAse III of SPCSV (SPCSV-R3). Relative gene expression of SPCSV-CP and SPCSV-R3 were not assayed in transgenic plants (R3-H and R3-F) and wild-type plants (Wt-H, Wt-F, Wt-C and Wt-FC), respectively. The actin housekeeping gene was used for RT-qPCR normalization. Data are shown as the mean ± 95% CI (n = 10–12)

Viral distribution in the plants grown at NaPPI was assayed in single-infected plants (Wt-C, Wt-F) and co-infected plants (Wt-FC). Symptoms of leaves in single- and co-infected plants varied (Fig. 3A). From the upper to lower leaves, accumulation of SPCSV increased in both plants, whereas SPFMV slightly decreased in co-infected plants (Wt-FC) and had very lower titres in single-infected plants (Wt-F) (Fig. 3B). Moreover, in co-infected plants (Wt-FC), accumulation of SPFMV in younger leaves was higher than SPCSV, whereas older leaves showed the opposite trend, with a cross-over point around leaf five in the study (Fig. 3B, black arrow). In addition, the level of accumulation of SPCSV in single-infected plants (Wt-C) ranged from 1.5 (leaf 2 and 3) to 54 (leaf 6 and 7) times that of co-infected plants (Wt-FC), whereas the opposite occurred for SPFMV with accumulation in single-infected plants (Wt-F) that was drastically lower than that in Wt-FC plants, with an average of 0.01 and 5.61, respectively (Fig. 3B).

Virus accumulation was assessed by RT-qPCR in the first fully developed leaves of all plants grown in NAPPI at 31 dpt. Viral accumulation was estimated by measuring relative expression of SPFMV or SPCSV coat protein, in addition to the relative expression of exogenous RNase III in R3-H and R3-F plants. Relative expression of exogenous RNase III in plants R3-F and R3-H was insignificant compared to expression of SPCSV and SPFMV (Fig. 3C, green bars). SPCSV accumulation did not significantly differ between single-infected (Wt-C) and co-infected (Wt-FC) plants (Fig. 3C, red bars). However, SPFMV accumulation showed a significant difference among single-infected (Wt-F), co-infected (Wt-FC), and transgenic infected (R3-F) plants, with relative virus accumulation of 0.0009, 15.976, and 4.316, respectively. It is noteworthy that accumulation of SPFMV was significantly different between transgenic plants infected with SPFMV (R3-F) and co-infected plants (Wt-FC) (Fig. 3C, blue bars).

### Effect of viruses on photosynthesis

**Fig. 4:**
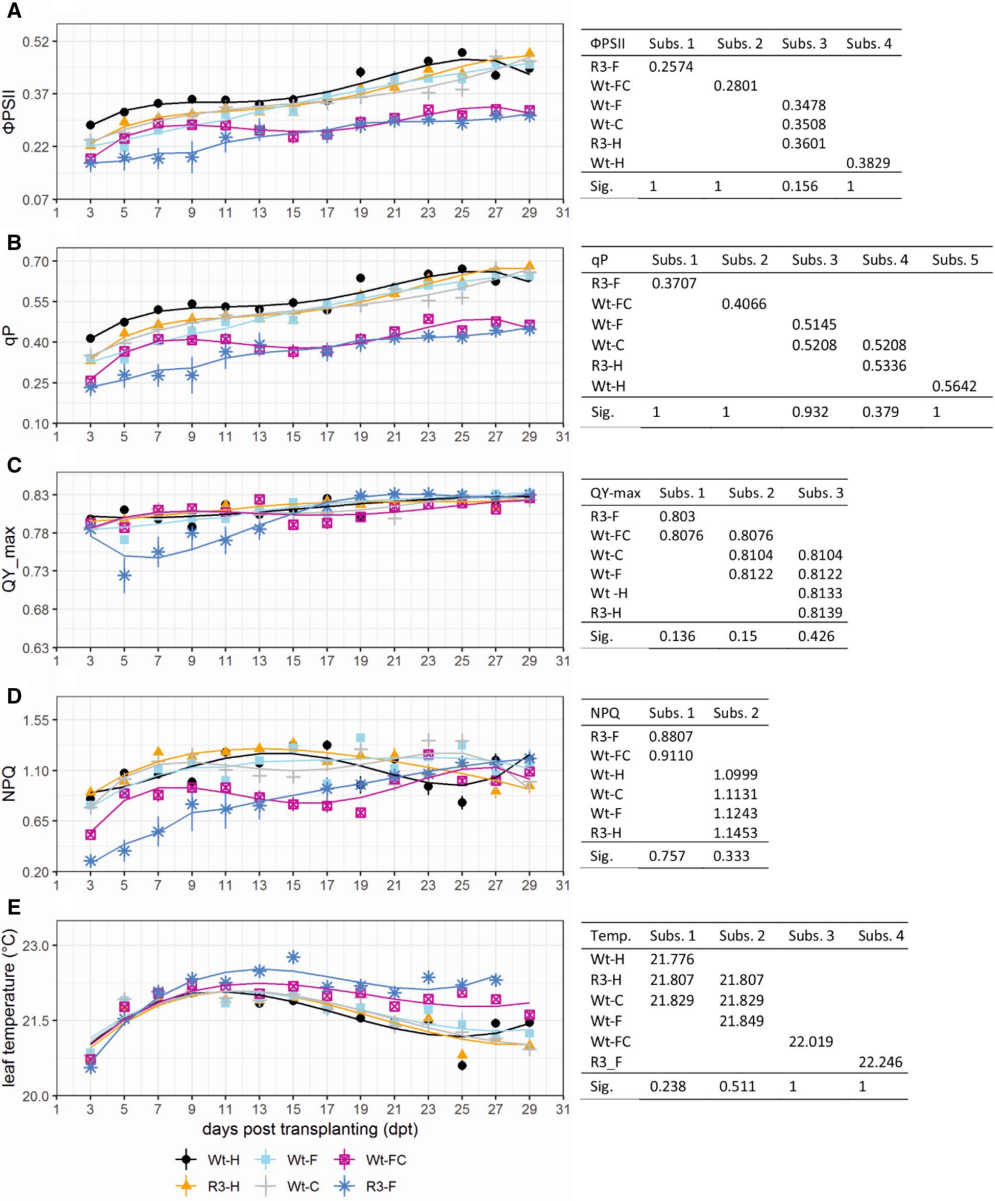
Effects of viral infection on PSII in sweetpotato plants were characterized by ChlF and TIR imaging. Graphs indicate observed data ± SE (n = 7–10) along with a line that shows the data-fitted model indicating the effect trend of viral infection over 29 dpt in wild-type and transgenic sweetpotato plants that were treated differentially. Tables on the right display data-fitted model mean values of the six groups clustered into different subsets (Subs.) by Tukey’s HSD test according to their significance (Sig.). **A**–**D**, the PSII response was assessed by ФPSII (**A**), qP (**B**), QY_max (**C**), and NPQ (**d**). **E** The leaf temperature effect was monitored by TIR imaging

To assess PSII performance, we focused our analysis on five PSII-related parameters, ФPSII, qP, QY_max, NPQ, and leaf temperature (Fig. 4). Other relevant parameters such as F0, Fm, Fv, and Fvʹ were also included (Additional file 1: Fig. S2).

ФPSII estimates the theoretical proportion of light used by chlorophyll associated with PSII [32, 33]. Variance analysis of ФPSII showed significant variation among the six viral treatments (Fig. 4A, left). Specifically, Tukey’s HSD test (Fig. 4A, right) showed that the six treatments could be grouped into four subsets. Wild-type plants infected with either SPCSV (Wt-C) or SPFMV (Wt-F) and transgenic healthy plants (R3-H) were grouped into the same subset (values were 0.3508, 0.3476, and 0.3601, respectively), meaning that their ФPSII values were not statistically different (P > 0.5). All other treatments including healthy (Wt-H), co-infected (Wt-FC), and transgenic infected (R3-F) plants were in their own subsets with mean values of 0.3829, 0.2801, and 0.2574, respectively. Altogether, our results demonstrated that a decrease in PSII photochemistry efficiency was correlated with the severity of disease symptoms.

qP is a measure of the proportion of open PSII reaction centres, indicating photochemistry capability. This value is nonlinearly correlated with the redox state of the quinone in most cases [34, 35]. In our study, qP varied significantly among viral treatments (Fig. 4B, left). Results from the Tukey’s HSD test were similar to those of ФPSII, except qP was able to distinguish transgenic healthy (R3-H) plants and plants infected with only SPFMV (Wt-F) and thus was the most sensitive parameter in our study. Specifically, we observed significant differences among viral treatments, with five subsets ranging from 0.3707 to 0.5642 (Fig. 4B, right). Moreover, the low values of qP for both co-infected (Wt-FC) and transgenic infected (R3-F) plants depict their inability to open their PSII reaction centres, hence limiting their ability to absorb light for photochemistry. Overall, statistical results from photochemical quenching and ФPSII were consistent, which was expected as these two parameters are usually positively correlated [36].

QY_max indicates the maximum efficiency at which light absorbed by PSII is used for reduction of the quinone [33]. QY_max was consistent across most of the viral treatments within 29 dpt. However, transgenic plants infected with SPFMV (R3-F) had much lower QY_max value and greater QY_max variability from 5 to 11 dpt (Fig. 4C, left). This may have resulted from the upward shift in parameters F0 and Fm during those days (Additional file 1: Fig. S2A, B). The Tukey’s HSD test showed that there was a significant effect of viral treatment on QY_max, the results from which were grouped into three subsets (Fig. 4C, right). Specifically, there were no significant differences between wild-type healthy (Wt-H), transgenic healthy (R3-H) plants, and the two groups of plants infected with only a single virus (Wt-C and Wt-F). In addition, no statistical difference could be found between single-infected and co-infected plants, or between co-infected plants and transgenic plants infected with SPFMV (Fig. 4C, right).

NPQ estimates the constant rate of heat-loss for PSII [37]. The overall time course of NPQ showed that most of the treatments resulted in values that were consistent with those from the previously described parameters. However, an unexpected decrease in NPQ in healthy plants (Wt-H) was observed from 23 to 25 dpt (Fig. 4D, left). The Tukey’s HSD test showed that there was a significant effect of viral treatments on NPQ (Fig. 4D, right). Specifically, one subset had a lower NPQ value (0.896 ± 0.015), indicative of an important heat-loss, and comprised the most severely symptomatic plants (Wt-FC, R3-F). The other subset, which included the remaining treatments, had a relatively high value (1.121 ± 0.016).

In addition, thermal imaging by visualizing leaf surface temperature is an effective method for detecting stress resulting from virus infection both spatially and temporally [11, 38]. Overall, the time course of leaf temperature showed that plants with the most severe symptoms (Wt-FC, R3-F) maintained a higher temperature, although all plants showed a slight decrease in temperature over time. In particular, differences in temperature among viral treatments increased slowly from 7 to 22 dpt (i.e., the data were more spread out), whereas there was no substantial difference during the first 7 dpt when leaves may have been too small to note a clear difference (Fig. 4E, left; Additional file 1: Fig. S3). However, wild-type healthy plants (Wt-H) and healthy transgenic plants (R3-H) showed a downward temperature shift at 25 dpt, which is consistent with our NPQ results. The Tukey’s HSD test indicated significant effects of viral infection on leaf temperature, resulting in four subsets among the groups (Fig. 4E, right). Specifically, we observed significant differences among healthy plants (Wt-H), plants infected with SPFMV alone (Wt-F) or co-infected with both viruses (Wt-FC), and transgenic plants infected with SPFMV (R3-F). Higher temperatures were associated with the severity of disease symptoms. In addition, no statistical differences could be found between single-infected (Wt-F or Wt-C) and transgenic healthy (R3-H) plants (21.632 ± 0.028 °C), or among wild-type healthy (Wt-H), SPCSV-infected (Wt-C), and transgenic healthy (R3-H) plants (21.579 ± 0.20 °C). Thus thermal imaging could be used as an indicator of the severity of viral infection in sweetpotato after only a week of growth. The temperature variation might be explained by a reduction in the proportion of PSII open reaction centres caused by viral infection [19].

### Gene expression related to photosynthesis

As we observed a clear reduction in PSII efficiency in all of our infected plants, we decided to assess the functionality of both photosynthetic and Calvin cycle pathways for single- and double-infected plants (Wt-F, Wt-C, and Wt-FC). To do so we monitored the fold change in gene expression as compared with that of Wt-H plants. Target genes consisted of three essential genes involved in PSII complex formation (*PsbA*, *PsbC*, and *PsbN*), one constitutive gene of the PSI complex (*PsaA*), both the large and small domain of Rubisco (*RbcL* and *RbcS1*), a regulator of Rubisco (*Rca*), and an essential component of glycolysis and gluconeogenesis (*FBA5*).

**Fig. 5:**
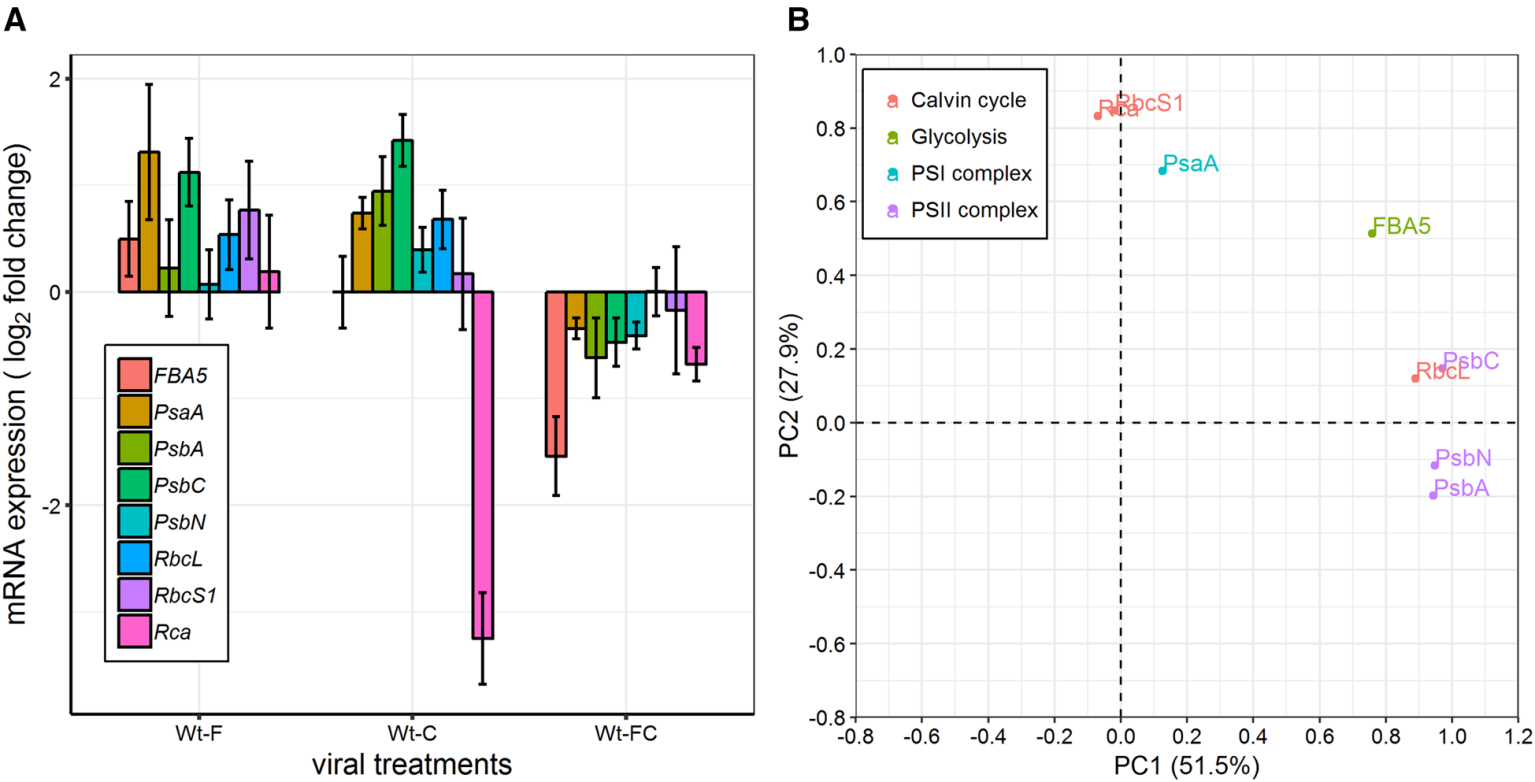
Fold change and PCA of genes expression. **A** The fold change in expression of wild-type sweetpotato infected with SPFMV (Wt-F) or SPCSV (Wt-C) or with both viruses (Wt-FC) was assessed relative to that of healthy plants (Wt-H). Data are shown as the mean ± 95% CI from seven sample pools representing 15 plants in total. **B** PCA of gene expression as in **A**. Percentages of total variance denoted in parentheses. Each color represents genes from a different pathway

Wt-C plants showed an upregulation of gene expression related to PSI and PSII complexes including *PsaA*, *PsbA*, *PsbC*, and *PsbN*. In Wt-F, only *PsaA* and *PsbC* were upregulated, whereas the expression of other PSII complex genes (*PsbA* and *PsbN*) was stable and comparable to that of healthy plants (Fig. 5A). Moreover, *FBA5* and *RbcS1* expression in Wt-C was similar to healthy plants but was upregulated in Wt-F. Rubisco regulator (*Rca*) underwent an abnormally strong downregulation in Wt-C, but in Wt-F its expression did not differ from that of healthy plants. In addition, *RbcL* was upregulated in both single-infected plants. For Wt-FC, expression of both PSI and PSII complex genes (*PsaA*, *PsbA*, *PsbC*, and *PsbN*) was downregulated, depicting a clear disruption of the photosynthesis pathway. PCA results showed that PSII complex genes (*PsbA*, *PsbC*, and *PsbN*) and the Rubisco large domain gene, *RbcL*, were grouped together (PC1 > 89%). *PsaA*, *RbcS1*, and *Rca* were grouped together (PC2 > 68%). *FBA5* was not present in those two groups and was explained by PC1 (76%) and PC2 (51%) (Fig. 5B).

In summary, most of the studied genes were upregulated in wild-type sweetpotato infected with SPFMV or SPCSV (Wt-F and Wt-C), whereas most of these genes were downregulated in co-infected wild-type plants (Wt-FC) (Fig. 5A). Taken together, these results indicated that viral synergism between SPCSV and SPFMV leads to a clear dysfunction in photosynthesis, glycolysis, and gluconeogenesis pathways in co-infected plants. Furthermore, the genes in those pathways were upregulated in single-infected plants with mild symptoms, which is possibly due to the interaction between plants and viruses.

## Discussion

To our knowledge, this is the first systematic study of SPCSV and SPFMV synergism in sweetpotato using ChlF and TIR imaging analyses. First, we compared two different growth conditions with different light quality and intensity. Despite the obvious growth difference between plants grown in the growth chamber and the NaPPI facility (Fig. 1A), relative viral accumulation and morphological changes were quite similar for the same viral treatment, indicating that accumulation and impact of SPCSV and SPFMV were not that sensitive to plant growth variation. Moreover, the shoot/root ratio of biomass showed no differences among our six viral treatments, except for R3-F plants, which were extremely symptomatic and small (Fig. 2B; Additional file 1: Fig. S1). This finding indicates that the effects of the plant-virus interaction are more systemic than local, which is consistent with SPFMV and SPCSV movement from plant vines to roots [39]. In addition, the shoot/root ratio and plant growth were significantly affected by the growth conditions. The shoot/root ratio was higher in NaPPI plants than in growth chamber plants (Figs. 1, 2A). There are two main hypotheses about plant biomass allocation, the balanced growth hypothesis and the allometric allocation hypothesis [40]. The former states that plant biomass is allocated proportionally to specific plant organs based on limiting resources, e.g., biomass allocation favours leaves if the light resource becomes limiting [41, 42]. The allometric allocation hypothesis, in contrast, stated that allocation occurs largely as a consequence of plant size [43]. Our data are in accordance with the balanced growth hypothesis. It is worth noting that a study on *Wheat streak mosaic virus* (WSMV) on hard red winter wheat showed that photosynthetic parameters, grain yield, and shoot biomass have a linear relationship [44]. It might be interesting to develop a sweetpotato-specific model that can predict underground biomass by looking at the aboveground biomass, as our data showed that infection with SPFMV and SPCSV does not affect the shoot/root ratio in wild-type sweetpotato plants.

The effect of SPFMV and SPCSV on sweetpotato development was monitored using several physiological and morphological parameters (Fig. 1). The RNase III transgene of SPCSV and single infection by SPFMV did not have significant effects on plant growth, as compared with Wt-H plants. In contrast, Wt-C, Wt-FC, and R3-F plants showed obvious symptoms. Our results are reasonably consistent with previous observations that synergistic effects are caused by the RNase III of SPCSV [8]. In addition, the accumulation of SPCSV and SPFMV was consistent with previous studies that showed a 600- to 1000-fold increase in SPFMV accumulation in co-infected plants, whereas accumulation of SPCSV was decreased as compared with respective single-infected plants [4, 45]. However, our data showed that accumulation of viruses can vary dramatically, especially for SPFMV (with a mean increase of 16,000 times in Wt-FC relative to Wt-F). Variation could also results from different quantification methods and the difficulty of precisely quantifying SPFMV in Wt-F plants with very low virus titres. The decreased accumulation of SPCSV in Wt-FC as compared with Wt-C might be caused either by active plant defences triggered by the increase in SPFMV or by a competition between the two viruses. It is also interesting to note that SPCSV alone had a significant impact on plant growth, whereas SPFMV did not, which is consistent with previous studies [8, 46].

Wt-FC showed more severe disease symptoms than did Wt-C. However, the absolute value of total virus accumulation (the sum of SPFMV and SPCSV) in Wt-FC was much less than the accumulation of SPCSV in Wt-C (only one-eighth the value over all leaves) (Fig. 3B). There are two reasons that might explain this difference. First, by taking into consideration viral expression modulation and symptom development between Wt-C and Wt-FC plants, the severity of viral symptoms in Wt-FC was most likely caused by SPFMV instead of SPCSV. Consequently, Wt-FC plants had more severe symptoms than Wt-C plants, which was caused by the proportion of SPFMV in Wt-FC, even if the total accumulation of both viruses was lower than SPCSV accumulation in Wt-C quantitatively. Second, viral accumulation localization was significantly different between SPCSV and SPFMV in Wt-FC plants, in which SPFMV was prone to locate in relatively young leaves, whereas SPCSV accumulated in older leaves (Fig. 3B). Possibly because young leaves contribute more to plant development than older leaves [47], the difference in viral accumulation between Wt-FC and Wt-C could partially explain the difference in symptom severity. All together, we assume that one or several viral proteins from SPFMV could also directly suppress plant development and induce severe symptoms.

RNase III of SPCSV increases the accumulation of SPFMV by suppressing the plants’ RNAi defence system of plants [8, 48]. However, in our study, RNase III transgenic plants infected with SPFMV (R3-F) showed more severe disease symptoms than did those co-infected with SPCSV and SPFMV (Wt-FC) in many aspects of growth (Fig. 1B, C). We note that the expression level of SPFMV in R3-F was significantly lower than that in Wt-FC, and the accumulation of RNase III in R3-F was insignificant compared with the accumulation of SPCSV in Wt-FC (Fig. 3C). This situation suggested that the constitutive expression of RNase III in transgenic plants has a greater impact than the endogenous RNase III from SPCSV during its synergistic infection with SPFMV. This could possibly be explained by a specific activation of the plant immune response by SPCSV viral proteins other than RNase III alone. Altogether, these results make the pathogenicity of SPFMV and SPCSV more complicated. To fully understand the molecular mechanisms behind the viral synergism between SPCSV and SPFMV, protein–protein interaction studies involving key host factors need to be carried out.

ChlF imaging analysis has become one of the most efficient methods not only to study photosynthetic machinery but also to monitor the physiological response to biotic and/or abiotic factors by measuring the ratio of fluorescence emission from photochemistry to heat dispersion in a non-destructive manner [33, 49]. Over the 29 day period in this study, ChlF-related parameters ФPSII and qP showed a general increase (Fig. 4). This might be explained either by the measurement method of the facilities, where, over time, the plants grew closer and closer to the cameras and light sources or by the change in the ratio of young to old leaves as the plants aged [50]. In addition, ФPSII and qP were correlated with physiological and morphological parameters of height, biomass, and leaf area among our six viral treatments (Figs. 1, 4). We noticed that more severe disease symptoms were associated with less efficient photosynthesis, which is consistent with the role of photosynthesis in carbon fixation and its contribution to plant growth [51]. Our results are consistent to some extent with the previous study, which showed that the decline in ФPSII was affected by the duration of *Pea enation mosaic virus* (PEMV) infection [52]. We noticed that single-infected plants (Wt-C and Wt-F) had no difference in photosynthetic efficiency-related parameters ФPSII and qP (Fig. 4A, B) relative to one another, but they did have a significant difference at the physiological and morphological levels (Fig. 1B–D), e.g., Wt-F plants were as tall as control plants, but Wt-C plants were obviously shorter. ФPSII and qP were, however, capable of distinguishing Wt-H from Wt-F plants (Fig. 4A, B). There are two possible explanations for this observation, either ChlF parameters are more sensitive than morphological parameters with respect to estimating viral infection, or healthy and SPFMV-infected plants could have different ratios of photosynthetic electron flow used for carbon assimilation and photorespiration [53], in which case different levels of photochemical quenching could lead to the same biomass assimilation.

Our results also showed that, in general, more severe forms of viral disease were associated with lower values of maximum quantum yield of PSII (QY_max) (Fig. 1B, 4C). Many studies have shown that QY_max is a good indicator of plant stress [54–57]. In addition, QY_max in the wild-type healthy sweetpotato (which is a C4 plant) was 0.814 ± 0.016, which is consistent with a previous study showing that C4 plants have a lower value but greater variation than C3 plants for QY_max (0.832 ± 0.004) [58]. Even though QY_max was not as sensitive as ФPSII and qP in this study, it was able to differentiate treatments according to the severity of viral symptoms in general. Thus, plants with different virus treatments might have similar photosynthesis potential as represented by QY_max when all reaction centres are open under dark-adapted conditions but a greater variation under actinic light conditions as reflected by ФPSII and qP. It is thus possible that severely symptomatic plants failed to absorb light and open their reaction centres as efficiently as asymptomatic plants.

The analysis of NPQ and leaf thermography are powerful tools for detecting early stress symptoms before damage caused by a pathogen becomes visible. In particular, leaf thermography has been used to study pathogenic processes including virus infection and spreading [11, 17]. An increase in leaf temperature has been observed 1 week before ChlF changes in infected leaves [59]. In our study, more severe viral symptoms were associated with higher leaf temperatures and NPQ values (Fig. 4; Additional file 1: Fig. S1). Changes in NPQ and leaf temperature in symptomatic plants might be a reflection of reduced transpiration and a decrease in stomatal density or increase in stomatal closure, which could be sequentially induced by the plants’ anti-viral defence process [59, 60]. Leaf thermography, as an indicator of heat dissipation (correlated with stress) is relatively straightforward as compared with NPQ measurements [11]. NPQ is correlated with changes in heat dissipation efficiency in the dark-adapted state, which is a photoprotection strategy of PSII when plants are exposed to intense light that overwhelms photosynthetic electron transport [61, 62]. We assume that viral infection induced a decrease in the stomatal aperture and transpiration rate leding to an increase in leaf temperature and further caused a decrease in photosynthetic efficiency, which was accompanied by an increase in NPQ. These networks and processes might all affect one another to reach an equilibrium during viral infection. However, the limitation of using NPQ and thermography is that those two parameters are inappropriate to directly distinguish virus stress from abiotic stress, especially when plants in the field are exposed to many environmental factors. Further laboratory tests including nucleic acid-based and serological-based methods are necessary to diagnosis viral disease at present.

Defence mechanisms are cost-intensive, and thus infected plants generally show a repression in carbon metabolism, chloroplast function, and photosynthesis [63, 64]. In this study we demonstrated that all selected genes involved in the photosynthetic, Calvin cycle, and glycolysis pathways were downregulated in co-infected plants. Consequently, the overall trend of our results indicated that chloroplast function and photosynthesis of sweetpotato were negatively impacted by co-infection with SPFMV and SPCSV, confirming our ChlF and TIR imaging and morphologic results.

In susceptible tobacco plants, the extent of downregulation of the photosynthetic pathway correlates with the severity of chlorosis symptoms induced by different CMV variants [65]. In our study, single-infected plants showed much less severe symptoms than did co-infected plants. However, with respect to gene expression related to the rate of photosynthesis, instead of an expected downregulation, we observed an upregulation of the photosynthesis, Calvin cycle, and glycolysis pathways in both single-infected plant groups (Wt-C, Wt-F). In potato plants, during the early stage of PVY infection, numerous photosynthesis-related genes are upregulated before their later downregulation [66]. It was suggested that photosynthesis-related genes are increased in response to elevated energy demands during the first response to stress. Thus, it is tempting to think that the presence of SPCSV or SPFMV in sweetpotato may cause a constitutive stress leading to the upregulation of genes in these pathways. Similarly, the *Grapevine rupestris stem pitting*-*associated virus* (GRSPaV) has evolved to a compatible interaction with *Vitis vinifera* without the development of phenotypic alterations, during which, interestingly, key genes involved in host photosynthesis also show strong upregulation [67]. Altogether, these findings support the idea of a possible activation of at least the photosynthetic pathways in the presence of asymptomatic or less-symptomatic viral stress. It is worth noting that, in sweetpotato, SPFMV failed to properly replicate and remained at a very low titre and SPCSV induced few symptoms even with relatively high titres, which might be sufficient to activate the photosynthetic pathways without triggering the full activation of plant defences which usually leads to the collapse of photosynthesis [68, 69]. Altogether, this could explain why single-infected sweetpotato were able to avoid and/or limit the formation of symptoms.

## Conclusion

Our analysis of virus-infected sweetpotatoes identified the most informative parameters for monitoring the severity of viral infection in this crop. These results could open a way to further investigate mechanisms implicated in physiological processes at the molecular level during viral infection of sweetpotato and provide some information for improving precision agriculture for sweetpotato.

## Materials and methods

### Plant material and growth conditions

Healthy sweetpotato plants of the cultivar Huachano (CIP42006) [2] were obtained from the germplasm collection of the International Potato Center (CIP) and side graft-inoculated with SPFMV (East African strain isolate Nam1), SPCSV-Ug (East African serotype 2), or both viruses as described in previous study [70]. Healthy and SPFMV single infected transgenic sweetpotato expressing SPCSV RNase III were previously obtained by Cuellar et al. [8]. Stem cuttings of each plant were used to generate six in vitro mother plants for the study: (a) ‘wild-type’ healthy sweetpotato (Wt-H), (b) sweetpotato infected with only SPFMV (Wt-F), (c) sweetpotato infected with only SPCSV (Wt-C), (d) sweetpotato co-infected with both SPFMV and SPCSV (Wt-FC), (e) healthy transgenic sweetpotato expressing SPCSV RNase III (R3-H), and (f) transgenic sweetpotato infected with SPFMV (R3-F). All mother plants were propagated and maintained in vitro on Sweetpotato medium [3 g l^−1^ MS salts, 30 g l^−1^ sucrose, 0.2 g l^−1^ ascorbic acid, 0.1 g l^−1^ l-arginine, 20 mg l^−1^ putrescine-HCL, 2 mg l^−1^ pantothenate calcium, 0.1 g l^−1^ calcium nitrate, and 4 g l^−1^ Gelride (Duchefa), pH 5.7]. In vitro plantlets were propagated by taking single-node stem cuttings. After cuttings developed newly formed roots on Sweetpotato medium, plantlets were transferred to pots (6 × 6 × 10 cm) fully filled with a mix out of 1/3 sand, 1/3 humus, and 1/3 washed soil and were then grown in the NaPPI facility and in a growth chamber with a light intensity of 260 and 60–70 µmol m^−2^ s^−1^, respectively. Both growing conditions had the same temperature (22 °C), humidity (60%), and photoperiod (16-h light/8-h dark). The number of monitored plants for each viral treatment in the NaPPI facility and growth chamber was 10 and 5, respectively.

### High-throughput plant phenotyping

The Plantscreen Conveyor System of the NaPPI platform at the University of Helsinki was applied to monitor and characterize plant viral disease symptoms. Measurements were obtained every second day over 29 days. A top-view photograph taken with an RGB camera (IDS Imaging Development Systems GmbH, Obersulm, Germany) was used to delineate plant surface area with Morpho Analysis 1.0.5.1 software (PSI, Brno, Czech Republic). ChlF imaging was obtained by a pulse amplitude modulation system (PSI) using a fluorescence camera (400–1000 nm). A quenching protocol to determine ChlF parameters was set up according to the ‘wizard’ included with FluorCam 7.0 software. Shutter and sensitivity were adjusted for our plant material to 33.33 µs and 5%, respectively. Thermal imaging was obtained with a thermal camera (7500–14,000 nm) FLIR A615 (FLIR Systems, Inc., Wilsonville, Oregon, USA). Temperature estimation and false-colour images were generated by Plantscreen Data Analyzer software (PSI). The whole high-throughput plant phenotyping experiment was carried out twice independently.

### Physical parameters of plants

Side-view photographs of all plants were taken manually after 7, 14, 21, and 28 days post-transplanting (dpt) using an EOS 760D camera coupled with an EF-S 17–85 mm lens (Canon, Tokyo, Japan). Plant height was measured using ImageJ software [71]. After 31 dpt, all leaves of > 2 cm in length were detached, and top-view images were taken as above. The total number of leaves were counted, and leaf area were calculated with Morpho Analysis software. At the same time, roots and shoots from each plant were separately harvested and immediately weighed to determine their fresh weight. Dry weight was measured after a 48-h incubation in an oven at 80 °C. The shoot/root ratio = (leaf dry mass + stem dry mass)/root dry mass, of each plant was calculated [29, 72].

### RNA isolation and RT-qPCR

To test viral accumulation in plants, samples (1 cm in diameter) from the 3rd or 4th fully developed leaf were collected at 31 dpt. Total RNA was extracted using the Spectrum Plant Total RNA kit (Sigma, St. Louis, USA). First-strand cDNA was synthesized from total RNA using Transcriptor First-Strand cDNA Synthesis kit with random hexamer primers (Roche, Basel, Switzerland). RT-qPCR was carried out using a LightCycler 480 Instrument II with LightCycler 480 SYBR Green I Master (Roche) and 10% of the newly synthesized cDNAs (2 µl cDNA, 5 µl Master mix, 2.5 µM primers) in a final volume of 10 µl. RT-qPCR was performed using the following cycling conditions: 95 °C for 10 min and 45 cycles of 95 °C for 10 s, 52 °C for 20 s, and 72 °C for 30 s, followed by a melting curve ramp from 95 to 65 °C. All RT-qPCR experiments were conducted in triplicate. For comparison of the data among samples, RT-qPCR results were normalized to the levels of the sweetpotato housekeeping gene *Actin* (EU250003.1) using specific primers (Additional file 1: Table S1) [73].

For virus titre experiments, primer pairs were designed by targeting the encoding region of SPFMV (NC_001841) coat protein (CP), of SPCSV CP (NC_004124), and of RNase III (GU127640). To test photosynthetic pathway regulation, we targeted four essential genes for PSII complex formation, *PsbA*, *PsbC*, *PsbE*, and *PsbN* (NC_026703), and *PsaA* which is a constitutive gene of the PSI complex (NC_026703). To test regulation of the Calvin cycle, we targeted both large and small domains of the Rubisco-encoding region (AY100962, LC036584); one of its regulators, *Rca* (EU287993); and an essential component of glycolysis and gluconeogenesis *FBA5* (KU166864). All primer pairs (Additional file 1: Table S1) were designed using NCBI primer BLAST tools.

### Data analysis

Effects of virus infection were estimated by the percentage reduction between plants from each infected condition and their corresponding control as follows: virus effect = (mean_infected_ − mean_control_)/mean_control_ × 100 [74]. Virus effects on height, biomass, and leaf surface area among the six treatment groups were analysed using one-way ANOVA and the post hoc Tukey’s honestly significant difference (HSD) test. Multiway ANOVA was used to analyse data from the shoot/root ratio of the six differentially infected plants growing under two conditions. TIR and ChlF data were examined using a general linear model for multivariate data. The differences among the six treatments for all parameters were compared using Tukey’s HSD with the significance level P = 0.05 to separate subgroups. All means from those groups in the homogenous subsets and multiple comparison tables are shown in “Results”. All ANOVA analysis was done with SPSS Statistics 25.0. To show trends in the raw data from TIR and ChlF imaging in detail, data were fitted with a generalized linear mixed model using lmer4 of R version 3.5.1.

Relative gene expression was calculated by the 2^−ΔΔCt^ method, as the efficiency of all primer pairs was close to 100% with a difference of < 5% among them (data not shown). Relative gene expression was also analysed by principal component analysis (PCA), in which Varimax with Kaiser Normalization was used as the rotation method. All figures were plotted by R package ggplot2 [75].

## Supplementary information

**Additional file 1: Table S1.** Primer sequences for RT-qPCR to detect viral accumulation and assess photosynthesis and the Calvin cycle pathway. **Fig. S1.** Representative photographs of plants from the six treatment groups under both growth conditions. **Fig. S2.** Effects of viral infection on PSII of sweetpotato plants as characterized by ChlF imaging. **Fig. S3.** Analysed top-view images of sweetpotato plants from the NaPPI plant phenotyping platform.

## Data Availability

All data generated or analysed during this study are included in this published article and its additional information files.

## Abbreviations

ChlF: chlorophyll fluorescence of PSII
F0: minimal ChlF yield
Fm: maximum ChlF yield
Fv: variable fluorescence
Fvʹ: variable fluorescence in the light-adapted state
NPQ: non-photochemical quenching
qP: photochemical quenching
QY_max: maximum quantum yield
SPFMV: *Sweet potato feathery mottle virus*
SPCSV: *Sweet potato chlorotic stunt virus*
TIR: thermal infrared
ФPSII: effective quantum yield of PSII
