# Supplementary material for: Phenotyping viral infection in sweetpotato using a high-throughput chlorophyll fluorescence and thermal imaging platform
Source: Plant Methods. 2019 Oct 22;15:116. doi: 10.1186/s13007-019-0501-1 (PMC6805361; doi:10.1186/s13007-019-0501-1)
Supplement: Supplementary file 1 — Additional file 1: Table S1. Primer sequences for RT-qPCR to detect viral accumulation and assess photosynthesis and the Calvin cycle pathway. Fig. S1. Representative photographs of plants from the six treatment groups under both growth conditions. Fig. S2. Effects of viral infection on PSII of sweetpotato plants as characterized by ChlF imaging. Fig. S3. Analysed top-view images of sweetpotato plants from the NaPPI plant phenotyping platform. [file 13007_2019_501_MOESM1_ESM.docx]

# Additional Table and Figures

**Table S1.** *Primer sequences for RT-qPCR to detect viral accumulation and assess photosynthesis and the Calvin cycle pathway*

| **Name** | **Sequence (5' to 3')** | **Size (bp)** |
| --- | --- | --- |
| *Viral Primers* | |  |
| FCP-F | ATCAAAGGGTGCGAGGGATA | 226 |
| FCP-R | GTCCCCTTTCACTCCCTCAT |  |
| CCP-F | TGTGAGGCAAATTTCAGGGG | 210 |
| CCP-R | ACGTCGGGCATATTGGATTG |  |
| CR3-F | TGCTGAATCTCTGTCGTTGC | 223 |
| CR3-R | ATGCACAACCAACCAACCAA |  |
| *Photosynthesis Pathway Primers* | |  |
| spPsbN-F | CAGCGACCCTAGTTACCATCTTTC | 119 |
| spPsbN-R | TCCATGTTCTTCGAATGGATCTCT |  |
| spPsbA-F | AGCCTATGGGGTCGTTTCTG | 236 |
| spPsbA-R | CCTATAGCTGCAGAAGTCGGA |  |
| spPsbC-F | CGCGCATTTGTATGGTCTGG | 227 |
| spPsbC-R | AAACCAGTAGGGCCTTGAGC |  |
| spPsaA-F | TGGATCACTTAGCCACGCTT | 201 |
| spPsaA-R | GGCCCCGATACTACCACTTG |  |
| *Calvin Cycle Pathway Primers* | |  |
| spRbcL-F | ACCGATGGACTTACCAGCCT | 181 |
| spRbcL-R | GCAGTGCTTTGAACCCAAATACA |  |
| spRbcS1-F | GTCCGCCGACTTTACTTCCATCT | 265 |
| spRbcS1-R | CAGCTTCCACATGGTCCAGTAT |  |
| spRca-F | GAGAAGGTGAACGAGAGGCTT | 193 |
| spRca-R | TAATGGCATCTGCGTTAGCGT |  |
| spFBA5-F | ATGACATCAAGAAATGCGCCG | 150 |
| spFBA5-R | CTTCTGCTGCAACCTTTGGGC |  |
| *Housekeeping Gene Primers* | |  |
| spACT_F | GTTATGGTTGGGATGGGACA | 199 |
| spACT_R | GTGCCTCGGTAAGAAGGACA |  |

**Fig. S1** Representative photographs of plants from the six treatment groups under both growth conditions. Side-view photographs of wild-type sweetpotato plants that were either healthy (Wt-H) or infected with SPFMV (Wt-F), SPCSV (Wt-C), or both viruses (Wt-FC), as well as transgenic sweetpotato plants expressing RNase3 of SPCSV that were either healthy (R3-H) or infected with SPFMV (R3-F). Plants were grown in the growth chamber (GC) or at the NaPPI facility (NaPPI) and were photographed at 31 dpt.

**Fig. S2** Effects of viral infection on PSII of sweetpotato plants as characterized by ChlF imaging. Measurements of PSII parameters were carried out over 29 dpt in wild-type and transgenic sweetpotato plants. Data are shown as the mean ± SE (n = 7−10). Tables on the right display data-fitted model mean values of the six groups clustered in different subsets (Subs.) by Tukey’s HSD test according to their significance (Sig.). (A−D), The response of photosystem II (PSII) was assessed by minimum fluorescence in the dark-adapted state (F0) (A), maximum fluorescence in the dark-adapted state (Fm) (B), variable fluorescence in the dark-adapted state (Fv) (C), and variable fluorescence in the light-adapted state (Fv’) (D).

**Fig. S3** Analyzed top-view images of sweetpotato plants from the NaPPI plant phenotyping platform. Top-view images of wild-type sweetpotato plants that were either healthy (Wt-H) or co-infected with SPFMV and SPCSV (Wt-FC) from 3 to 17 dpt. Photographs were obtained using the three imaging systems from the NaPPI facilities: visible-light image (RGB); ChlF represented by variable fluorescence in the dark-adapted state (Fv), photochemical quenching (qP), and effective quantum yield of PSII (ФPSII); and TIR imaging. False-color images of ChlF parameters display a heat map color scale from dark blue to red ranging from 0 to 700 for Fv and from 0 to 1 for qP and ФPSII, whereas the TIR parameter is represented by a heat map color scale from blue to red ranging from the lowest to the highest temperature.


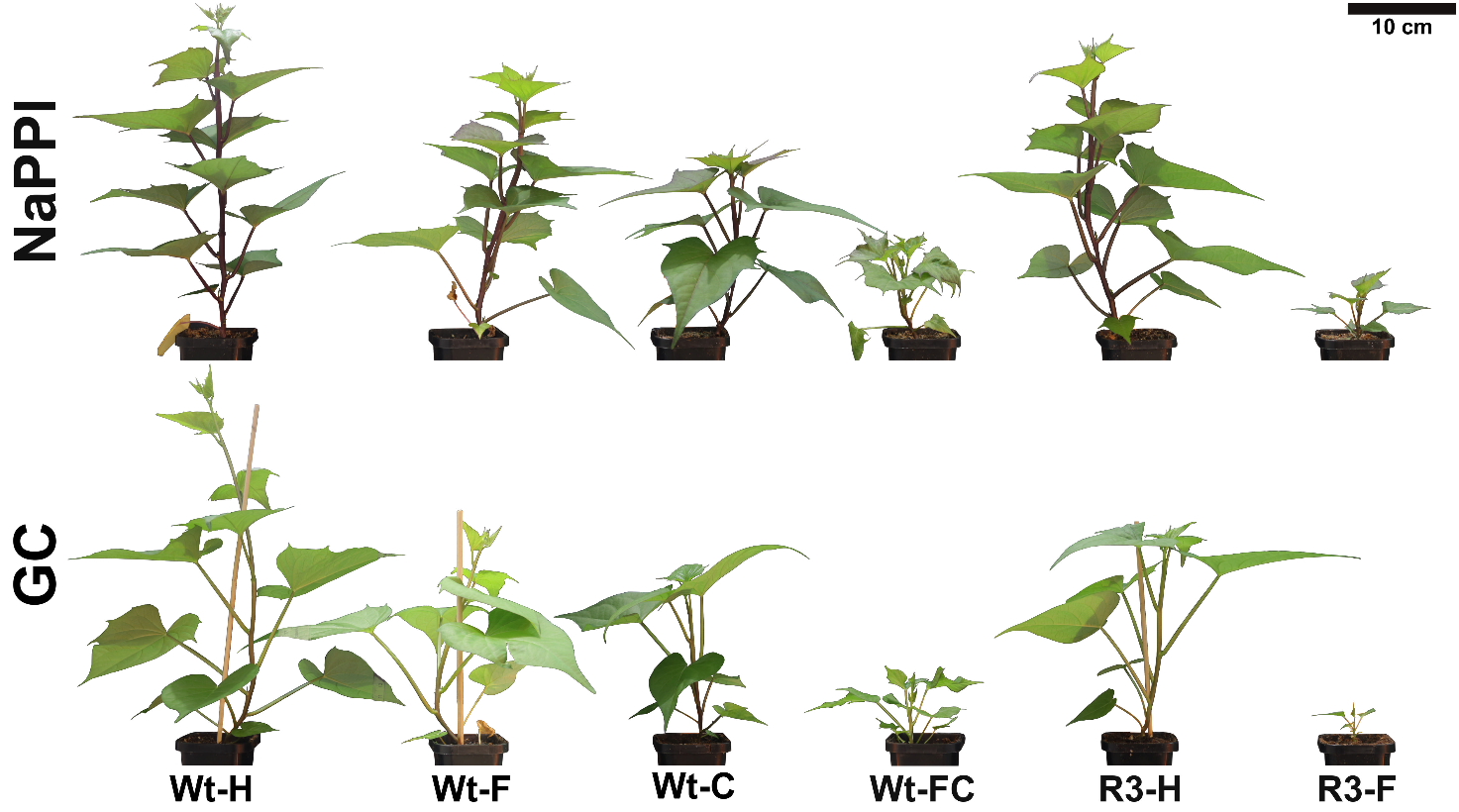


**Fig. S1**

**
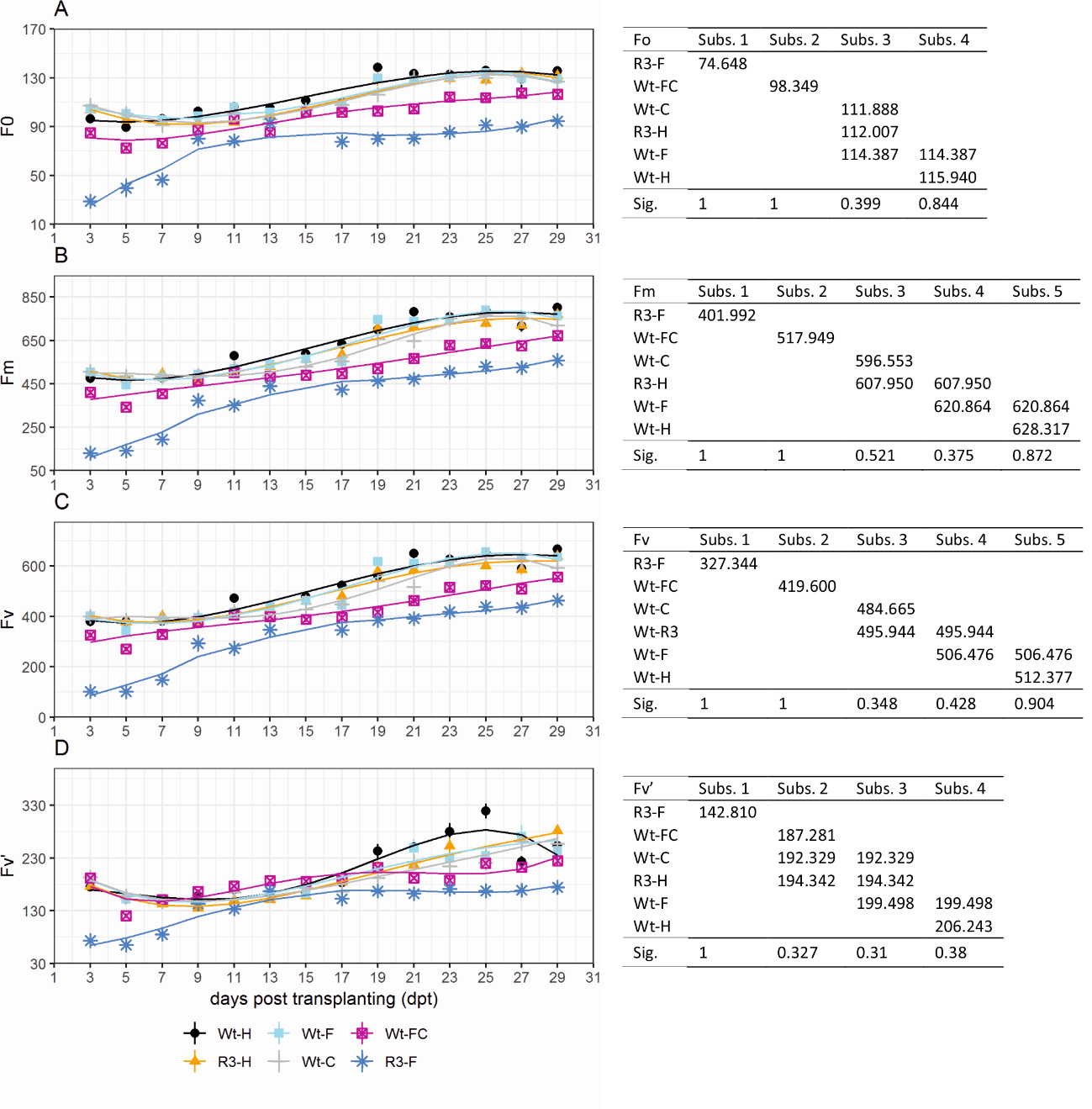
**

**Fig. S2**


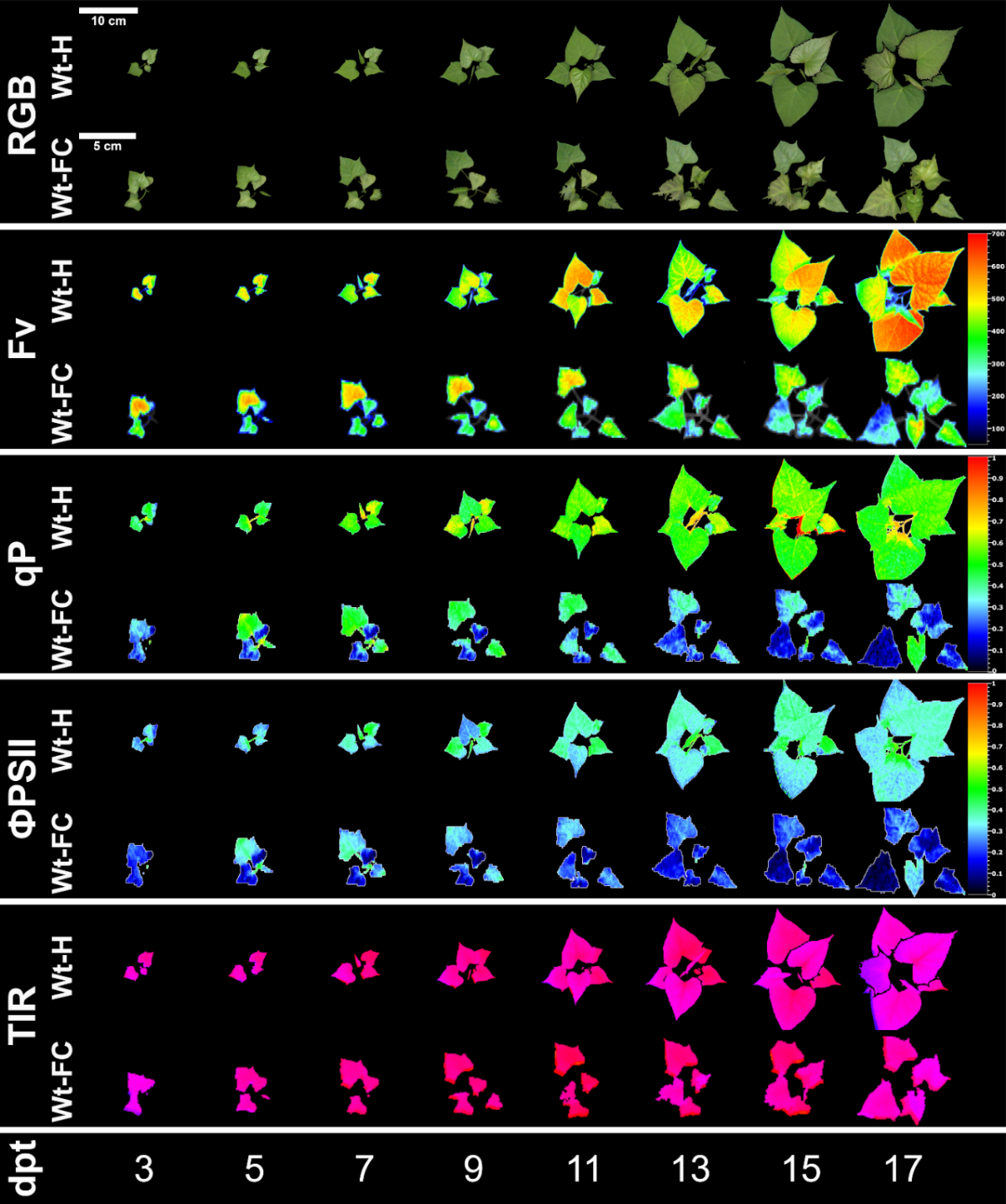


**Fig. S3**
